# HPA Axis in the Pathomechanism of Depression and Schizophrenia: New Therapeutic Strategies Based on Its Participation

**DOI:** 10.3390/brainsci11101298

**Published:** 2021-09-30

**Authors:** Joanna Mikulska, Gabriela Juszczyk, Monika Gawrońska-Grzywacz, Mariola Herbet

**Affiliations:** Chair and Department of Toxicology, Faculty of Pharmacy, Medical University of Lublin, 8b Jaczewskiego Street, 20-090 Lublin, Poland; joannamikulska12856@gmail.com (J.M.); gabajul@wp.pl (G.J.); mariola.herbet@umlub.pl (M.H.)

**Keywords:** HPA axis, depression, schizophrenia, cortisol, inflammation, stress, psychiatric disorders

## Abstract

The hypothalamic-pituitary-adrenal (HPA) axis is involved in the pathophysiology of many neuropsychiatric disorders. Increased HPA axis activity can be observed during chronic stress, which plays a key role in the pathophysiology of depression. Overactivity of the HPA axis occurs in major depressive disorder (MDD), leading to cognitive dysfunction and reduced mood. There is also a correlation between the HPA axis activation and gut microbiota, which has a significant impact on the development of MDD. It is believed that the gut microbiota can influence the HPA axis function through the activity of cytokines, prostaglandins, or bacterial antigens of various microbial species. The activity of the HPA axis in schizophrenia varies and depends mainly on the severity of the disease. This review summarizes the involvement of the HPA axis in the pathogenesis of neuropsychiatric disorders, focusing on major depression and schizophrenia, and highlights a possible correlation between these conditions. Although many effective antidepressants are available, a large proportion of patients do not respond to initial treatment. This review also discusses new therapeutic strategies that affect the HPA axis, such as glucocorticoid receptor (GR) antagonists, vasopressin V1B receptor antagonists and non-psychoactive CB1 receptor agonists in depression and/or schizophrenia.

## 1. Introduction

The hypothalamic-pituitary-adrenal (HPA) axis plays an important role in the body’s adaptation to stressful situations [1,2]. HPA axis malfunction occurs in many mental diseases, including depression and schizophrenia. A relationship has been shown between disorders caused by stressful stimuli, especially long-term ones, and depression. Childhood traumatic events related to stress factors significantly increase the risk of mental illness in adulthood. The mechanism of this phenomenon is based, among others, on HPA axis dysfunction. Moreover, it was shown that arginine vasopressin (AVP) is co-secreted with corticoliberin (CRH) related to the HPA axis [1,2]. HPA axis hyperfunction observed in depression and stress seems to be a significant therapeutic problem [3]. The normalization of the HPA axis activity in patients with depression may prove to be an effective target of pharmacotherapy and understanding the exact mechanisms of their synergistic action may contribute to the development of new therapeutic strategies for this disease [3].

Schizophrenia is one of the most mysterious conditions in the medical world [4,5,6]. Current treatment is aimed at alleviating the symptoms of the illness rather than a causal cure. Despite much research into schizophrenia, its etiology is still not fully understood. In recent years, increasing attention has been paid to the importance of the HPA axis and gut-brain axis in the pathogenesis of schizophrenia [4]. One of the possible pathways leading to the development of schizophrenia seems to be the activation of inflammation associated with chronic stress. Elevated levels of C-reactive protein, the pro-inflammatory transcription factor NF-κB and IL-6 have been observed in such patients [4]. Chronic stress can lead to neurodevelopmental disorders and dysregulation of the HPA axis. It should be noted that IL-6 activates the HPA axis at every level [4]. Severe stress experienced in childhood (as in the case of depression) increases the chance of developing schizophrenia [5]. An imbalance between the antioxidant defense system (AODS) and the production of reactive oxygen species (ROS) leads to oxidative stress. An imbalance between these systems is observed in psychotic disorders, such as schizophrenia [6]. The research presented in recent years demonstrates the complexity and intricacy of the pathological processes of schizophrenia. Exploring new mechanisms of disease onset may enhance personalized therapy and represents a potential target for future research theories [4,5,6].

## 2. HPA Axis Regulation

**Figure 1:**
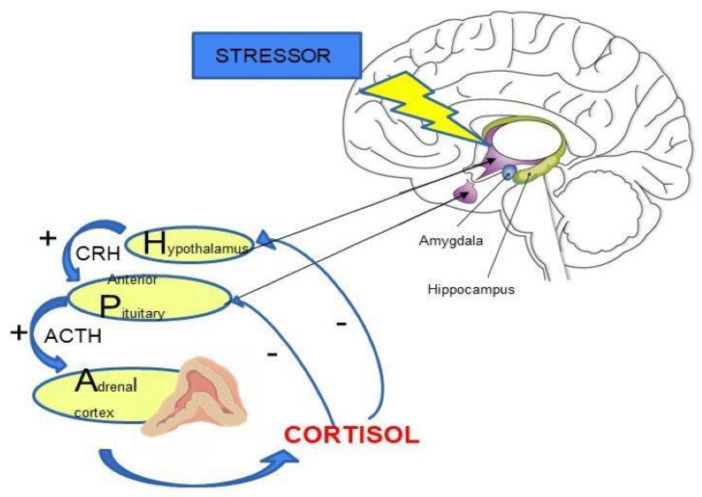
Regulation of hypothalamic-pituitary-adrenal (HPA) axis activity: stress as a factor activating the HPA axis.

The HPA axis plays a key role in maintaining body homeostasis and the body’s response to stress. Stress results in the release of corticoliberin-releasing hormone (CRH) from the hypothalamus. This information is then transmitted to the anterior lobe of the pituitary gland, where the secretion of adrenocorticotropic hormone (ACTH) takes place. This leads to stimulation of cortisol release into the blood from the adrenal cortex [7,8]. Increased cortisol level leads to inhibition of CRH and ACTH secretion by a negative feedback loop [7,9] (See Figure 1).

**Figure 2:**
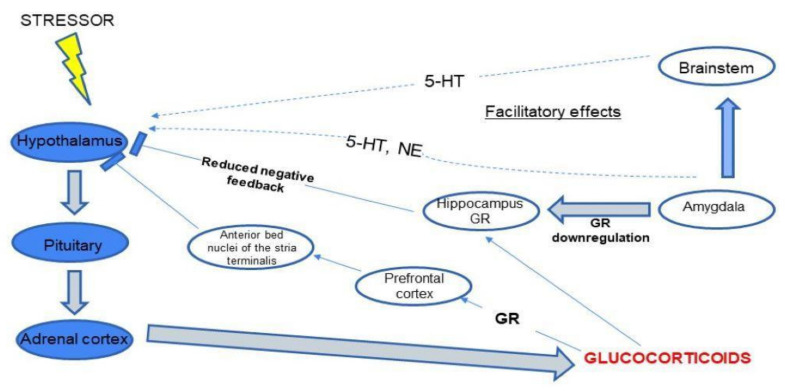
Activation of the HPA axis including the role of the amygdala, hippocampus and prefrontal cortex. The amygdala stimulates the HPA axis through NE and 5-HT neurotransmission and attenuates the negative feedback exerted by glucocorticoids, leading to a decrease in GRs in the hippocampal area. Glucocorticoids stimulate (through GR) the prefrontal cortex which attenuates the negative feedback to HPA-axis.

The importance of the HPA axis is mainly based on the action of cortisol (See Figure 2). Cortisol is secreted in stressful situations as a defense response of the body. It reduces the inflammatory response, stimulates gluconeogenesis and is responsible for protecting the body from an excessive immune response [10]. Activation of the HPA axis also occurs in non-stress-related situations. This is related to the regulation of circadian rhythms, e.g., the highest cortisol levels are observed in the morning [11,12].

Some research suggests that increased cortisol secretion may lead to cognitive impairment [12,13,14]. Stawski et al. [15] observed the correlation between the level of cortisol and cognitive function. The decreased cognitive functioning (among participants of the clinical trials, was associated with a lower level of cortisol. Constant exposure to stressors disrupts proper cognition, resulting in irreversible changes to the neuronal system [16]. Factors affecting the availability of steroids to mineralocorticoid (MR) and glucocorticoid (GR) receptors may influence the action of glucocorticosteroids in the central nervous system (CNS). Numerous studies confirm the extra-genomic regulation of HPA axis function by glucocorticoids. Hyperactivity of the HPA axis in depression results from impaired negative feedback mediated by the GR [17]. Medial hypothalamic neurons have been shown to be responsible for inhibiting CRH and vasopressin release [18]. Endocannabinoids are involved in this process. The binding of endocannabinoids to CB1 receptors in the brain leads to the inhibition of neurotransmitter release in the prefrontal cortex and probably in the hippocampus. Moreover, the constant exposure to stressors disrupts the signaling CB1 receptors in the amygdala which finally activates the HPA-axis [19]. The increasing level of GR leads to the dysregulation of the neuronal system. This suggests the involvement of the HPA axis in the pathogenesis of neuropsychiatric disorders [18,20]. Data from animal models [21] indicates that the endocannabinoid system is involved in mechanisms of anxiety. Various stressful situations activate different receptors which not only release various neurotransmitters but also activate the immune system and plastic mechanisms [21,22]. Dysregulation of the HPA axis is also strongly associated with the pathogenesis of depressive disorders [9,23].

Interestingly, there is also research suggesting that the effects of the serotonergic system and the HPA axis overlap [24]. This is indicated by the fact that serotonin, while regulating mood, also affects the activity of the HPA axis. Moreover, chronic activation of the HPA axis leads to an increase in the number of serotonergic (5-HT) receptors in the hippocampus, amygdala and frontal cortex. Serotonergic neurotransmission and the HPA axis function modulate neuronal plasticity, including hippocampal neurogenesis. This may explain the increased incidence of depression in older people who develop cognitive impairment [24].

It has been postulated that the development of depression reflects dysregulation of HPA axis function. More than 40–60% of patients experience hypercortisolemia or other HPA axis disorders [24]. Increased cortisol levels are also associated with cognitive impairment. The effect of CRH on ACTH release is strongly enhanced by vasopressin. Additional activation of CRH neurons is observed in depressive disorders, among others. This may be related to aversive stimuli or due to the presence of genetic factors [23,24].

## 3. The Role of Cortisol in Depression and Schizophrenia

Cortisol hypersecretion is associated with acute and more severe subtypes of major depressive disorder MDD; however, the enhanced concentration of this hormone is noticeably absent in the atypical subtype [25,26]. This is supported by several studies conducted on depressed patients [26,27,28,29]. Cortisol hypersecretion is caused by the stress response [30]. In chronic mild stress (CMS) models, elevated corticosterone levels have been observed in response to stress, and inhibition of corticosterone synthesis has been associated with inhibition of depressive behavior in CMS [31,32]. Cortisol hypersecretion may also contribute to the development of other disorders, such as psychosis in MDD and results from increased dopaminergic activity [33]. One of the first studies in animal models, which focused on analyzing the correlation between stress-induced elevated cortisol levels and the development of depression, found a significant association [34]. Researchers also determined cortisol levels in the hair of rhesus monkeys as a biomarker of chronic stress [35,36]. The association between the observed increase in cortisol levels and the development of depression may be due to the fact that excessive adrenal activity leads to a destructive effect on the hippocampus and increased vulnerability to depression [34,35,36]. Chronic stress plays a large role in the development of depressive disorders and it is involved in elevated cortisol levels, which is largely due to dysregulation of the HPA axis [37,38,39]. Hypercortisolism is one of the important mechanisms involved in response to stress [37]. Patients suffering from MDD had significantly higher stress and cortisol levels than control subjects [37]. This is associated with the development of glucocorticoid resistance and an increase in depressive symptoms [40,41].

The relationship between stress, cortisol and the development of depression can also be investigated by exogenous administration of corticosterone (CORT) in animal models [42]. This method appears to be effective due to the lack of predictability of the stress stimuli, which may contribute to inconclusive results [43]. Many studies indicate that prolonged administration of CORT contributes to the development of depression-like behaviors (such as learned helplessness and anhedonia) in the forced swimming test, which is dose- and time-dependent [44]. It has been observed in both animals and humans that repeated administration of CORT leads to cognitive dysfunction and some hippocampal-dependent memory tasks [45]. Exogenous administration of CORT has made it possible to visualize neurobiological changes in the brain that occur during depression, such as reduced hippocampal volume [46], atrophic changes in the prefrontal cortex and hypertrophy of the amygdala dendrites [42,47]. There have also been studies that have observed that paradoxically not only chronic stress but also physical exercises elevated basal cortisol levels, however, the effects on stress coping, cognition and memory were different [48]. They were detrimental in case of chronic stress and beneficial during physical exercises [48]. Physical exercise may regulate cortisol secretion in response to stressful stimuli. Moreover, voluntary exercise has been shown to improve memory and stress coping in test animals [48,49,50]. Dopamine (DA) is a catecholamine that is responsible for improving cognitive function and memory. It was proved that the reward system, whose neurotransmitter is DA, is activated in test animals in response to exercise. Interestingly, a reduction in DA neurotransmission is observed in depression caused by chronic stress [51,52]. Research also suggests that the medial prefrontal cortex plays an important role in the negative feedback regulation of the HPA axis. Exogenous administration of CORT to the medial prefrontal cortex decreased plasma ACTH levels in response to acute stress [51]. The link between CORT and DA levels is confirmed by the fact that the administration of a GR antagonist to the midbrain, leads to a reduction in DA levels in the medial prefrontal cortex [53].

Abnormal functioning of the HPA axis, in the form of cortisol hypersecretion, can exacerbate symptoms of psychiatric disorders [54]. Hair cortisol concentration (HCC) allowed for the estimation of cumulative cortisol secretion over a few months [55]. It was proved that HCC is linked to the severity of delusions in patients suffering from schizophrenia [55]. Therefore, HPA axis dysfunction may be one of the biomarkers of schizophrenia [54]. There are also studies showing significant sex differences in cortisol levels. Indeed, high cortisol levels were observed in women with schizophrenia compared to a control sample, while no significant changes were observed in men [56]. HPA axis dysfunction in patients with schizophrenia is associated with an impaired response to psychosocial stressors [57,58,59]. Chronic stress is a contributing factor to the inflammatory response in schizophrenia. This is confirmed by the observed increase in blood IL-6 levels [57]. Hyperfunction of the HPA axis in response to stress-induced inflammation may occur differently than in healthy volunteers [58,59]. One of the most recent studies was focused on investigating cortisol/DHEA ratios in people with schizophrenia [60]. This is also the first research to link the cortisol/DHEA ratio with reduced brain volume in patients suffering from this mental disease. It was shown that reduced cortisol/DHEA ratios observed in schizophrenic patients were inversely correlated with patients’ volumes of the hippocampus as well as the dorsolateral prefrontal cortex. Thus, the findings suggest that the cortisol/DHEA ratio may be a biomarker of the loss of brain tissue, in particular hippocampal and cortical damage, as well as HPA axis dysfunction in schizophrenia [60]. Studies have also shown elevated serum DHEA levels and no differences in cortisol concentration in comparison to healthy control [60]. It may result from compensatory responses to the stress and increased inflammation found in schizophrenia [61]. DHEA has neuroprotective effects that are more pronounced in stressful situations [62].

## 4. HPA Axis and Gut Microbiota

There is growing evidence suggesting an impact of the gut microbiome on the HPA axis. It is thought that the microbiota may influence animal behavior via the microbiota-gut-brain axis. Studies in mice have shown that the microbiota influences the body’s response to stress [23]. Studies on the effects of probiotic intake indicate an important role for the microbiota in regulating stress and emotional responses [63]. To investigate how the microbiota affects brain function, scientists examined hormone levels in the HPA axis in mice. The mice were divided into two groups (germ-free (GF) and specific pathogen-free (SPF)). After an in-depth analysis of the results, it was found that SPF mice displayed more anxiety-like behaviors in response to the same stress stimulus. In addition, GF mice showed large differences in hormone levels in the HPA axis compared to SPF [19]. Gut microbes can affect the HPA axis through the endocrine system, which then leads to overactivity of the HPA axis [64,65]. Although there is still no conclusive data on the influence of the microbiota on anxiety behavior, more and more research is being conducted in this area. The experiments performed by researchers at Chongqing Medical University showed that mice lacking hormone receptor genes and mice given hormone receptor antagonists modulated their behavior by adapting to a stressful stimulus [66]. This led to increased expression of GR and MR receptors [67]. Alterations in these receptor levels are associated with HPA axis dysfunction, and increased expression of GR receptors is observed in depression. Furthermore, high intestinal permeability and inflammatory mediators are important factors in mental disorders [68]. A disturbed gut microflora leads to impaired neurogenesis. Metabolites formed in the intestine are able to cross the blood-brain barrier and affect the functioning of the central nervous system [69,70]. The gut microbiota has been shown to influence CNS function through:

- Enterochromaffin (EC) cells, which control serotonin (5-HT) synthesis [70,71];
- Humoral pathways (via microbiota metabolites and gut hormones);
- Activation of immune responses via the vagus nerve [72];
- Production of short-chain fatty acids (SCFAs) in the gut, which affects the balance of microglia and leads to the release of intestinal peptides, influencing the activity of the brain-gut axis [73].

The gut microbiota can also influence the metabolism of tryptophan, which then forms serotonin, and also produces dopamine, γ-aminobutyric acid and acetylcholine, affecting the stability of the HPA axis [74]. Dietary supplements, such as n-3 polyunsaturated fatty acids (PUFAs) may prevent the development of depression by inhibiting HPA axis hyperexcitability, as confirmed by a preclinical study in rats.

A growing body of scientific research is pointing to bidirectional links between the gut microbiota and the HPA axis. The link between the gut microbiota and the neuroendocrine system is also supported by related disorders, such as depression and irritable bowel syndrome (IBS) [65,75,76]. Activation of the HPA axis is able to change the composition of the microbiota, which has been reported in patients with depression. Increased intestinal permeability and a disturbed microbiota composition can lead to neuroendocrine disorders [77].

Stress in early childhood significantly affects the HPA axis activity. Over-activity of the HPA axis leads to resistance of immune cells to the anti-inflammatory effects of cortisol. Stress also affects the composition of the gut microbiota, which can occur through the activation of neuroendocrine hormones [75,77,78]. HPA axis hyperreactivity is associated with excessive CRH secretion and impaired glucocorticoid hormone action [73]. There are studies that suggest that activation of the HPA axis by the gut microbiota may result from increased permeability of the intestinal barrier and gut microbiota-induced inflammation [79]. Furthermore, lipopolysaccharide (LPS), a component of the outer cell membrane of Gram-negative bacteria, is able to cross the intestinal epithelial barrier leading to activation of the HPA axis [79,80]. The gut microbiota is able to convert indigestible intestinal fibers into SCFAs which have a positive effect on the maturation of microglia cells, as confirmed by studies in mice [81]. Furthermore, systemic administration of butyric acid (which belongs to SCFAs) has been shown to exert antidepressant and neuromodulatory effects [81,82]. The microbiota not only affects the release of various intestinal peptides (peptide YY, glucagon-like peptide 1) but is also involved in their production (*Bifidobacterium dentium* are able to produce γ-aminobutyric acid (GABA)) [83,84]. In order to investigate the role of the microbiota on HPA axis function, the researchers conducted a study using isolation-cultured mice (GF) [85]. The mice studied had reduced GR expression in the cerebral cortex and increased plasma levels of ACTH and corticosterone in response to stress [85]. Colonization with enteropathogenic *Escherichia coli* enhances the HPA stress response [85]. Changes at the neurohormonal level were observed in GF mice. Among other things, they showed reduced expression of NMDA and BDNF receptor genes in the hippocampus, which can lead to long-term CNS damage [86].

One of the first studies on the functioning of the microbiota-gut-brain axis [87] showed that stress in adulthood modifies the composition of the gut microbiota. Indeed, chronic stress leads to the translocation of bacteria across the intestinal barrier [87]. The composition of the intestinal microbiota is also altered. Most noticeable was an increase in *Clostridium* spp. and a decrease in *Bacteroides* spp. A study in laboratory animals also demonstrates the important role of the gut microbiota altered by stress on immune function [87]. The mechanisms by which this occurs are not fully understood; the sympathetic nervous system, which is involved in the transmission of signals between the brain and the gut, may play an important role [87]. Interestingly, a study in mice [88] showed that the composition of the gut microbiota does indeed have a significant effect on the memory of the animals studied. Mice exposed to acute stress also showed memory impairment [88]. Therefore, modifying the composition of the gut microbiota may be a promising therapeutic target for improving memory processes [88].

## 5. The Neurobiology of Depression: Possible Pathophysiological Mechanism Including HPA Axis

Biological and psychosocial factors are inextricably linked to the genesis of depression [89]. One of the features of major depression disorder (MDD) is the dysfunction of the HPA axis. Long-term depression is a much more serious condition than episodic depression and can lead to the development of comorbidities. Such patients often develop social phobia, which has an adverse effect on the progress of the illness. A deep understanding of the neurobiology of depression may hold the key to effective treatment. With the advent of neuroimaging techniques, magnetic resonance imaging (MRI), positron emission tomography (PET) and functional fMRI, the areas of the brain responsible for controlling emotions and behavior have been identified [90].

The prefrontal cortex (PFC) constitutes the highest level of the cortical hierarchy dedicated to the representation and execution of actions. It is divided into three main parts: the dorsolateral frontal cortex, the orbitofrontal cortex (OFC), and the medial frontal structures (including the anterior cingulate cortex) [91]. The ventromedial cortex (VMPFC) is essential for generating emotions. It participates in the regulation of impulses coming from the autonomic and neuroendocrine systems, modulating pain and aggression. The PFC, the amygdala and the hippocampus play an important role in the development of depression [92]. PET studies showed abnormalities in regional cerebral blood flow in several structures of the prefrontal and limbic cortex [90].

**Figure 3:**
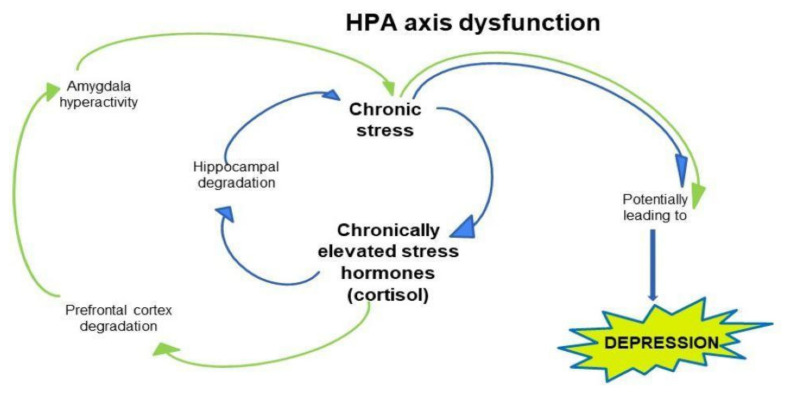
HPA axis dysfunction: role of amygdala and hippocampus in neurobiology of depression.

The amygdala is involved in regulating cortical arousal and responses from the neuroendocrine system to emotional stimuli. Abnormal function of the amygdala correlates with the severity of depression (See Figure 3).

A meta-analysis of MRI studies showed that the size of the amygdala is reduced in patients with untreated depression [93]. Morphological changes of the amygdala in depression have been confirmed in several studies. In contrast to the hippocampus, increased expression of brain-derived neurotrophic factor (BDNF) was observed [93]. BDNF plays a key role in regulating the plasticity of stress-induced synapses. Depression also leads to impaired glutaminergic signaling in the amygdala. The volume of the amygdala changes with the development of depression [93]. Furthermore, increased amygdala activity induced by negative stimuli may be an early marker of depression risk [93,94]. However, the abnormal functional connectivity in depression is divergent in the left amygdala, where functional connectivity has decreased [95]. Frontal-limbic circuits associated with the amygdala may change exponentially with the progression of depression. These circuits may provide biomarkers to study the effect of treatment on depression.

Many scientific studies confirm the huge role of the hippocampus in the neurobiology of depression. This is because the hippocampus is involved in learning and memory, the process of neurogenesis and is a structure rich in corticosteroid receptors. The hippocampus is closely linked to the hypothalamus, which is part of the HPA axis [96]. The role of the hippocampus is important for the regulation of the HPA axis. Changes in its degree of plasticity can result from stress, which affects hippocampal function in a number of ways [97]. Stress can reduce neuronal plasticity in the hippocampus and lead to activation of the HPA axis and consequently increased corticosteroid levels [97] (See Figure 3).

Hippocampal plasticity in depression refers to changes in hippocampal volume, inhibition of neurogenesis and neuronal apoptosis [97]. Stress significantly affects neuronal plasticity. *Lycium barbarum* has been shown to reduce depressive behavior mediated by increased plasticity of synapses in the rat hippocampus. Moreover, 5-HT1A receptors that are normally highly expressed in the hippocampus are silenced in animal models of depression [98]. A study by Murialdo et al. investigating the relationship between hippocampal dysfunction and the HPA axis found that elevated levels of dehydroepiandrosterone (DHEAS) indicated impaired hippocampal function in depression [99].

Plasticity of the synaptic hypothalamus in depression may be due to increased mRNA expression of synaptotagmin I and synapsin I. This contributes to the hyperactivity of the HPA axis and the development of depressive-type behavior [95,100]. A study by Jin-Frang Ge et al. [100] proved that exposing rats to unpredictable stress for a further 3 weeks can induce depressive-type behavior, including anhedonia. The stress factor leads to the synthesis and release of CRH by the paraventricular nucleus (PVN) of the hypothalamus. The hormone then binds to anterior pituitary receptors, leading to the release of ACTH. Eventually, ACTH triggers the synthesis of glucocorticoids [99,100]. Neurobiological changes in the regulation of cortisol secretion by the HPA axis are observed in MDD. Several meta-analyses indicate that depressed patients have elevated daytime cortisol levels compared to controls. Most studies support the fact that HPA axis disturbances are a cause rather than a consequence of the disease [101]. Many components of the HPA axis contain leptin receptors. The leptin pathway influences the stress response at each major level of the HPA axis. By acting on HPA axis structures and the hippocampus, leptin can exert antidepressant effects [89,102]. The substrate for serotonin synthesis in the central nervous system is tryptophan. A meta-analysis showed that tryptophan levels were reduced in depressed individuals compared to healthy controls [103]. Tryptophan 2,3-dioxygenase (TDO) and indoleamine 2,3-dioxygenase (IDO) are enzymes involved in tryptophan metabolism. Both in vitro and in vivo studies have shown that glucocorticoids cause activation of TDO, leading to reduced tryptophan levels. Increased levels of pro-inflammatory cytokines (IL-6, TNF, high-sensitivity C-reactive protein), which induce IDO activity, are observed in depressed patients [103,104]. The researchers examined tryptophan levels in the kynurenine pathway and its effects on HPA axis function. The study showed increased cortisol levels in the evening, which was due to a reduced kynurenine/tryptophan (kyn/trp) ratio. A study found an association between HPA axis function and tryptophan degradation in depression [89,104]. The results suggest that in depression, disruption of the HPA axis may be responsible for the formation of a new balance between endogenous cortisol levels and metabolism of tryptophan [89,105].

Postpartum depression (PPD) affects up to 20% of mothers. The molecular changes in the brain that occur in this type of depression are still not understood. Disturbed function of the HPA axis has been observed in postnatal female mice exposed to chronic stress. That was proved by increased levels of stress-activated cortisol and elevated CRH 1 expression in the medial hypothalamic nucleus (PVN) [106]. Impaired suppression of the HPA axis in women with PPD has also been observed. In response to stress or circadian rhythms, PVNs release CRH together with arginine vasopressin into the hypothalamic-hypothalamic circulation. CRH and AVP activate the pituitary-adrenal axis, leading to glucocorticoid secretion. Excessive glucocorticoid secretion leads to memory impairment and decreased hippocampal volume in test rats [106]. Chronic psychosocial stress during pregnancy (CGS) has been found to cause somatic changes in mothers and offspring and leads to dysregulation of the maternal HPA axis. Dysregulation of the HPA axis and inappropriate maternal behavioral responses are associated with altered expression of steroid receptors. The mechanism of maternal HPA axis suppression protects the body from elevated glucocorticoid levels. The chronic psychosocial stress paradigm during pregnancy (CGS) appears to prevent attenuation of the maternal HPA axis [107]. CGS disrupts adaptive changes in the maternal neuroendocrine system by altering maternal HPA axis activity. Increased CRH signaling in the PVN after CGS exposure was associated with a reduction in the number of nuclear steroid hormone receptors GR, PR and MR. These receptors regulate CRH expression. Chronically elevated corticosterone impairs HPA axis feedback inhibition through GR desensitization. Interestingly, stimulation of CRH neurons in the PVN has been shown to be sufficient to generate depression-like behavior. Increased CRH signaling of the PVN is associated with the appearance of abnormal postnatal behavior in the mother [108,109,110].

AVP is a cyclic nonapeptide that exists in two versions in the brain. One is responsible for regulating blood pressure and water balance, while the other is responsible for regulating the HPA axis. AVP is secreted by the PVN into the hypothalamic-pituitary portal circulation. Because of the close relationship between anxiety and depression, AVP is thought to mediate both diseases [111,112]. Moreover, AVP increases CRH activity leading to the release of ACTH from the anterior pituitary and consequently increasing HPA axis activity. Therefore, targeting the AVP system may contribute to new therapeutic strategies for the treatment of depression and anxiety. AVP influences stress responses in humans by increasing the cortisol response to environmental stressors [113].

## 6. Potential Antidepressants—Modulating Overactivity of the HPA Axis

HPA axis abnormalities include persistently elevated free cortisol levels in serum and urine, suppressed ACTH release and elevated CRH. It has, therefore, been hypothesized that HPA axis hyperreactivity may be a potential treatment target in depression. Current antidepressants are not effective in all patients, suggesting that monoamine depletion is not the only pathomechanism of depression. In addition, commonly used treatments lead to many side effects, such as sexual dysfunction, seizures, and cardiotoxicity. This makes it necessary to develop new drugs that are safer and more effective for the patient [114]. Many clinical and preclinical studies suggest that epigenetic mechanisms may play an important role in the treatment of depression [115]. New therapeutic options focus on the modulation of the HPA axis, BDNF, reduction of inflammatory cytokines and oxidative stress, and thyroid hormone action [115].

Polyphenolic phytochemicals may be used to treat geriatric depression as they modulate HPA axis hyperactivity and stimulate serotonergic neurotransmission. Phytochemical-based treatments for depression can be rapidly moved into clinical trials due to their good safety profile and high bioavailability. Preliminary results have shown that treatment with resveratrol (RES) can alleviate depressive symptoms. Resveratrol is a natural polyphenol whose action is mainly based on the regulation of the HPA axis. The over-reactivity of the HPA axis seen in depression leads to excessive corticosterone secretion. Researchers created a model of depression in rats by subjecting them to chronic unpredictable mild stress (CUMS) and the activity of the HPA axis was studied. Treatment with resveratrol has been shown to reduce serum corticosterone levels in test rats [114,116,117]. Many studies confirm that BDNF levels are reduced in depressed individuals [118]. Decreased BDNF expression in the hippocampus and prefrontal cortex in depressed patients has also been observed [118]. RES leads to increased BDNF production from astrocytes [117,118]. In the rat models studied, the increased mRNA expression levels of synaptotagmin I and synapsin I were also observed in the hypothalamus [116]. These proteins are involved in the pathophysiology of depression. Resveratrol may also alleviate symptoms of depression by increasing levels of the synaptotagmin I protein in the hippocampus. Recent studies have shown that resveratrol also inhibits monoamine reuptake and significantly reduces anxiety and depressive behaviour [116]. However, additional studies are needed to confirm its effectiveness in supporting the treatment of depression [116].

Another polyphenol with potential antidepressant properties is ferulic acid (FA), which shows strong anti-inflammatory properties. Preclinical studies were conducted in which FA (12.5, 25, and 50 mg/kg/day, i.g.) was administered for 28 days. Ferulic acid significantly reduced IL-6, IL-1β and TNF-α concentrations. These data suggest that the antidepressant effect of FA is due to decreased secretion of pro-inflammatory cytokines. Reduced concentrations of ACTH and corticosterone were also observed, through increased expression of GR [117]. Treatment with FA significantly reduced depression-induced nNOS expression in the hippocampus. Furthermore, FA inhibits depression-induced NF-κB activation in the hippocampus of offspring rats [117,119].

A growing body of research indicates that the retinoic acid receptor α (RARα) activates the HPA axis and is closely linked to the pathology of depression. RARα antagonists are being investigated as potential antidepressants. The use of Ro41-5253 (RARα antagonist) in preclinical models attenuates depressive behavior in rats, as evidenced by increased sucrose preference in sucrose preference test (SPT), reduced immobility time and increased swimming time in forced swimming test (FST), increased number of transitions and swings in the open field test (OFT). Decreased serum corticosterone levels indicate inhibition of HPA axis stimulation by Ro41-5253 [120]. In addition, Ro41-5253 increases the expression of BDNF and synapse-related proteins including postsynaptic density protein 95 (PSD95), synaptophysin (SYP). More research is needed to understand the antidepressant mechanism of Ro41-5253 [120,121].

In animal models studies it was proved that neuropsychiatric disorders in newborns may be caused by infections that are transmitted during the prenatal period of life and are associated with impaired regulation of the HPA axis [122]. Numerous preclinical and clinical studies have shown that minocycline, an antibiotic of the tetracyclines group, has an antidepressant effect by regulating the action of the HPA axis. Minocycline is currently being investigated as a potential antidepressant [123]. HPA axis dysregulation is central to the etiology of anxiety and depression. Nowadays, there is a growing body of research providing evidence that infection/inflammation early in life may be a risk factor for the development of neurodegenerative conditions. To investigate this, researchers administered lipopolysaccharide (LPS) to mice as an inflammation inducer. Administration of LPS led to anxiety and depression in the rodents [123,124]. Walker et al. showed that LPS activates microglia cells and increases pro-inflammatory cytokines in the brain. Studies have shown that dysregulation of inflammatory processes may be a very significant pathophysiological mechanism of major depression. LPS-induced inflammation was reversed after minocycline administration. Reduced production of pro-inflammatory cytokines was observed in the mice tested [125]. Minocycline also acts on the neurogenesis process in the hippocampus, resulting in an anxiolytic effect [125,126]. The neuroprotective mechanism of minocycline is probably due to its direct anti-inflammatory action. In addition, minocycline significantly increases neurogenesis in the hippocampus, which plays a key role in anxiety and depression and in the functioning of the HPA axis [125]. Furthermore, other studies using minocycline indicate its potential use in the treatment of schizophrenia. The rodents studied showed improvements in positive and negative symptoms, measured by the Scale for Assessment of Negative Symptoms (SANS) [127].

Recent preclinical studies in rodents suggest that vasopressin V1B receptor antagonists are effective in the treatment of depression [128]. Their action is based on attenuation of the HPA axis hyperactivity. This appears to be a promising treatment target for depression with HPA axis disorders [128]. VB1 receptors are synthesized in the brain, mainly in the hypothalamus and limbic system. These areas are responsible for the regulation of stress and emotions, which is why an increased expression of the VB1 receptor is observed in chronic stress. The arginine-vasopressin receptor (AVP) subtype is involved in the HPA axis regulation. Increased vasopressin production is seen not only in various areas of the brain but also in the blood, which may be associated with the increased risk of suicide. There is a strong correlation between VB1 receptor activation and ACTH secretion [128]. Moreover, the correlation between AVP and ACTH is stronger than that between CRH and ACTH. The effect of CRH and ACTH release is enhanced by vasopressin. There is ample evidence suggesting a role for the AVP-VB1 receptor system in depression. AVP levels are also elevated in brain areas, such as the PVN and supraoptic nucleus of the hypothalamus and the suprachiasmatic nucleus. This is also confirmed by preclinical studies in rats. Rodents in which AVP deficiency was observed exhibited reduced depressive and anxiety-like behavior [128]. The antidepressant effect of V1B receptor antagonists is due to inhibition of the HPA axis activated by acute stress. Preclinical studies in models of anxiety and depression are currently underway using molecules TASP0233278 and TASP0390325 acting as potential antidepressants through V1B receptor antagonism [128]. The effects of other VB1 receptor antagonists were also analyzed in preclinical and clinical models. In chronic stress models, SSR149415 exerted antidepressant effects similar to conventional antidepressants. Studies showed that SSR149415 prevented the elevation of ACTH levels triggered by stress. Injection of SSR149414 into certain brain nuclei showed antidepressant effects. SSR149415 reduced neurodegeneration in the hippocampus induced by chronic stress, leading to attenuation of depressive and anxiety-like behaviors. Furthermore, receptor antagonists for vasopressin inhibit acetylcholine (ACh) release in the hippocampus, similar to classical antidepressants, which may play a key role in their anxiolytic and antidepressant properties. The current study suggests that SSR149415 may serve as a useful tool to further investigate the effects of the HPA axis on the development of depression [128]. Clinical trials with VB1 receptor antagonists have shown them to be effective at doses that attenuate HPA axis overactivity. The greatest efficacy is observed in patients with HPA axis hyperreactivity in MDD. However, further studies are needed to confirm the role of the hippocampus in the antidepressant effects of VB1 [129,130].

Mifepristone (RU-486) is a GR receptor antagonist. Following administration of the drug, GR receptor counts increase rapidly, indicating compensatory activation of the HPA axis. Mifepristone administration can effectively alleviate depressive symptoms by increasing GR regulation in critical structures of the hypothalamus and limbic system. This leads to increased negative feedback control of GR over the HPA axis. In the studies on animal models, the efficacy of the drug is confirmed by the results of the forced swimming test. It was observed that repeated administration of mifepristone (5 days, 10 mg/kg) reduces immobility and increases swimming behavior. Treatment with mifepristone normalizes the stress-induced reduction in hippocampal neurogenesis [131]. A study conducted in the University of Cincinnati shows that the inhibitory effect of the hippocampus on HPA axis activity occurs through the ventral subiculum [132]. By increasing neurogenesis in this area of the hippocampus, mifepristone may inhibit stress-induced over-reactivity of the HPA axis [133]. Mifepristone has also been studied in other psychiatric conditions, including depression in schizophrenia. Clinical studies show that it suppresses HPA axis activity in people with severe mental illness. In addition, the administration of mifepristone led to a decrease in diurnal ACTH levels. Interestingly, the therapeutic effects of mifepristone were not observed until 14 days after cessation of treatment [131,134,135]. However, further studies focusing on the long-term effects of mifepristone on the HPA axis are needed to establish the efficacy of mifepristone in schizophrenia with depression [134,135].

Recent preclinical studies have demonstrated the efficacy of histone deacetylase (HDAC) inhibitors. Targeting histone acetylation appears to be a promising treatment for depression. Long-term stress leads to increased levels of acetylated histones in nucleus accumbens (NAc). Prolonged intake of HDAC inhibitors may contribute to neuronal plasticity by reducing HDAC2 expression in the NAc. This is the structure responsible for the regulation of depressive behavior [136,137]. A representative of HDAC inhibitors is vorinostat (VOR). Injection of this drug into the cerebellum of a mouse exposed to stress led to an antidepressant response [138,139]. In addition, VOR showed anti-inflammatory effects, suppressed iNOS expression in the induced inflammatory pathway and inhibited iNOS phosphorylation [138].

## 7. The Neurobiology of Schizophrenia: Possible Pathophysiological Mechanism Including HPA Axis

Stress is one of the main factors contributing to the development of psychiatric disorders. Chronic stress is associated with, among other things, excessive activation of the HPA axis. The HPA system, otherwise known as the stress axis, acts with some delay—after about 30 min [140]. CRH secreted by PVN cells into the pituitary portal circulation stimulates the secretion of ACTH into the blood. The final step in the activation of the HPA axis is the activation of adrenal cortex cells and the resulting secretion of glucocorticosteroids (cortisol). The delayed effect of the HPA axis is explained by the binding of glucocorticosteroids to nuclear receptors. After binding to the appropriate receptors in the cytoplasm, glucocorticosteroids must first be transported to the nucleus where they influence gene expression (GRE, glucocorticoid-response element) [140].

Regulation of the HPA axis plays a key role in the stress response. An initial increase in cortisol concentration in the blood has a beneficial effect on the body, but a prolonged increase in its concentration is harmful and appears to play a large role in the pathogenesis of psychiatric disorders [141,142]. Schizophrenia is a complex illness influenced by both genetic and environmental factors. Increased susceptibility to environmental stress in childhood has been shown to lead to hyperreactivity of the HPA axis in response to acute stress. Increased cortisol secretion may, therefore, contribute to the development of psychosis [143]. Long-term exposure to elevated blood cortisol levels has a strong effect on dopaminergic neurotransmission. There are several theories as to why schizophrenia develops. One of these is the dopaminergic concept. According to this theory, over-activity of the mesolimbic dopaminergic pathway is responsible for the development of positive symptoms (psychoses) [143]. In schizophrenia, there is also a decrease in dopaminergic activity in the prefrontal cortex, in the mesocortical pathway, which leads to the tracing of cognitive functions (negative and cognitive symptoms) [142,143]. Studies using positron emission tomography confirm that elevated cortisol levels lead to increased dopamine release in the striatum [144].

Despite the popularity of the dopamine concept, the endocannabinoid system (ECS) plays a large role in the development of schizophrenia. Cannabinoid receptors type 1 and 2 (CB1 and CB2) are metabotropic, G-protein related receptors. The best-known agonist of these receptors is delta-9-tetrahydrocannabinol (Δ9-THC), a *Cannabis sativa* alkaloid. CB1 receptors are found in large quantities in the mesolimbic pathway involved in the regulation of the HPA axis. In addition, CB1 receptors regulate glutamate release from synapses responsible for cortisol release [145]. Exogenous cannabinoids have been shown to increase the HPA axis activity. Injection of THC into test rats led to an increase in plasma corticosterone and ACTH levels [145,146]. Cannabinoids may exhibit their effects mainly by affecting corticosterone negative feedback at the hypothalamic level. Confirmation of this relationship in further studies may be crucial in accurately understanding the pathogenesis of psychiatric disorders, as well as depression, as over-activation of the HPA axis leads to changes in endocannabinoid synthesis. The endocannabinoid system exhibits neuroprotective effects and mitigates the effects of chronic stress [145]. The endocannabinoid system influences stress management, as evidenced by animal behavioral tests, including the forced swimming test. Studies have shown that mice with blocked CB1 receptors undergoing chronic stress exhibited marked anhedonia [145]. The HPA axis plays a key role in the response to chronic stressful stimuli. Long-term stress can cause psychological, metabolic and immunological disorders. This is why adaptation to chronic stress is necessary to prevent adverse effects of stressors. This is supported by the fact that in the absence of adaptation to stressful stimuli, CNS dysfunction occurs. Many studies indicate that the endocannabinoid system inhibits the HPA axis activity [147,148]. It has recently been noted that the inhibitory effects of the endocannabinoid system may be due to its action within the amygdala. This is supported by the fact that local administration of CB1 receptor agonists to this brain area can reduce the HPA axis activity, whereas administration of the same CB1 receptor ligands to another brain area does not have the same effects [149,150]. Furthermore, it is thought that ECS signaling may affect the HPA axis by inhibiting glucocorticoid-induced negative feedback. The endocannabinoid system plays an important role in the regulation of the HPA axis under chronic stress conditions, acting as a “guardian” of the HPA axis and preventing its over-activation [147].

A number of studies indicate adverse effects of both elevated and decreased cortisol levels on cognitive function. Cortisol secretion in the HPA axis is regulated by, among others, the hippocampus. It has been shown that hippocampal volume is significantly reduced in patients with schizophrenia and MDD compared to controls, with larger differences observed in patients with MDD [151]. This may be due to changes in hormone secretion in response to stress. The difference between schizophrenia and MDD may be related to the fact that HPA axis dysfunction is more important in the pathogenesis of diseases associated with mood disorders [151,152]. The relationship between HPA axis activity is still not entirely clear. However, there is a growing body of scientific work supporting the fact that HPA axis hyperfunction may enhance dopaminergic neurotransmission, which plays a key role in the pathophysiology of schizophrenia. It is believed that hypercortisolemia may be the cause of the onset of psychiatric disorders. Interestingly, studies show significant differences between the activity of the HPA axis in men and women, and thus, different degrees of mental illness incidence depending on gender. In male rodents, higher cortisol levels were observed in response to stressful stimuli compared to female individuals [153,154]. Abnormal functioning of the HPA axis may also be related to epigenetics. It has been shown that traumatic childhood events can lead to mutations in GR genes [155]. Abnormalities of the HPA axis may be related to the immune system. It has been shown that patients with psychosis have elevated blood levels of cortisol as well as inflammatory markers, such as IL-6, and IL-1-beta [156].

Stress is an important contributor to psychosis. Furthermore, it has been shown that patients with psychiatric disorders also have increased HPA axis activity, manifested by increased cortisol secretion [156]. The increased HPA axis activity is particularly prevalent at the onset of illness. This is also confirmed by the fact that individuals with a predisposition to psychosis have an increased pituitary volume [156]. The role of HPA axis hyperactivity in the pathomechanism of schizophrenia is also indicated by the fact that glucocorticoid antagonists are effective in relieving psychotic symptoms [157]. Numerous studies also indicate suppression of the HPA axis activity by atypical antipsychotics (AP) [158]. However, it has still not been shown whether elevated cortisol levels are responsible for the development of schizophrenia. Further research is needed to clarify the role of the HPA axis in the development of psychiatric disorders. A better understanding of the role of the HPA axis in the pathogenesis of schizophrenia, but also of cognitively related diseases, such as MDD, may be the key to developing new chemical molecules that modulate the HPA axis activity [157,158].

## 8. New Possible Drugs for Schizophrenia, Targeting the HPA Axis by Psychoactive Substances

Despite significant advances in the availability of pharmacotherapy for schizophrenia, the effectiveness of drugs is still limited. An increasing number of scientific studies targeting the HPA axis as a potential site of action for new therapies are emerging [157,159]. The HPA axis may play an important role in the pathogenesis of schizophrenia. Elevated blood cortisol levels are thought to contribute to cognitive decline in older people. This effect leads to activation of GR and inhibition of neurogenesis. The functioning of the HPA axis is largely regulated by GR and MR, therefore, it is believed that treatment targeting these receptors may have a beneficial effect on cognitive processes in schizophrenia. Neurosteroids, which include pregnenolone (PREG) and dehydroepiandrosterone (DHEA), appear to be a promising treatment option [134,157].

DHEA is included in the group of steroid hormones produced by the adrenal cortex. It is also produced in the brain and gonads acting through the GR. Recent studies have shown high concentrations of DHEA in the human brain, resulting from the ease of penetration of the DHEA molecule across the blood-brain barrier [160]. DHEA has a beneficial effect on neuronal growth, showing neuroprotective effects. The mechanism of action of DHEA is complex and subject to numerous regulations. One mechanism of action is based on genomic effects, whereby DHEA is converted to dihydrotestosterone and testosterone [161]. The resulting metabolites bind to androgen receptors, thus showing their pharmacological effects. However, this is not the only mechanism of DHEA action. This steroid may also combine with NMDA, nicotinic, glutamate or vanilloid receptors TRPV (transient receptor potential vanilloid channels) [162]. Several studies indicate that DHEA is effective in patients with schizophrenia in improving cognitive function. However, further studies using DHEA in schizophrenia are needed due to inconclusive results [163,164,165]. One of the intermediate products in the synthesis of DHEA is PREG, which is formed from cholesterol. It has been shown that pregnenolone can inhibit the negative symptoms of schizophrenia. This effect is probably due to its action on GABA-A, NMDA, sigma-1 and dopaminergic receptors [165,166]. PREG may improve cognitive function and reduce stress levels. In addition, some preclinical studies have shown that PREG alleviates cognitive symptoms in schizophrenia. Furthermore, this neurosteroid has a good safety profile [167]. Both PREG and DHEA have neuroprotective activity, protecting neurons from apoptosis, as confirmed by preclinical animal studies [168]. Some studies indicate that DHEA protects hippocampal cells from the damaging effects of cortisol. Interestingly, the use of classical antipsychotic drugs leads to increased DHEA levels in schizophrenic patients. The effect of neurosteroids in schizophrenia is still not fully understood and requires further research [168].

The inefficiency of treatment with classical antipsychotics is a serious problem for patients, which is why the search for new pharmacological therapies is so important. ECS plays an important role in the maintenance of normal mental health, so more and more potential treatments for schizophrenia targeting ECS are being developed. Some naturally occurring cannabinoids may find use as APs. An increasing number of studies indicate that ECS inhibits HPA axis activity by affecting CRH in the PVN and ACTH secretion from the pituitary [169]. Furthermore, the mechanism of ECS inhibitory action on the HPA axis is due to its effect on glucocorticoid secretion. Glucocorticoid binding to GR in the PVN leads to the release of endocannabinoids, which by binding to presynaptic CB1 receptors lead to inhibition of glucocorticoid release [170].

To date, there has been little research on the effects of psychoactive substances on schizophrenia. These compounds may be used by patients with schizophrenia to reduce feelings of fear and anxiety. The use of psychoactive substances may relieve symptoms of the illness in the short term, but long-term use is associated with permanent changes in the brain. There is then a reduction in volume in the limbic system and prefrontal cortex [170]. Psychoactive substance abuse is a serious problem in modern society and can lead to serious health consequences. Numerous studies confirm the fact that psychoactive substances significantly increase the activity of the HPA axis, leading to excessive cortisol secretion. Increased levels of ACTH and β-endorphins have also been observed in users of psychoactive substances, such as cocaine. The effect on CRH secretion is thought to play a major role. This is supported by the fact that the administration of a CRH antagonist abolishes the effects of cocaine on the HPA axis. However, a full understanding of their pharmacological and toxicological effects is necessary to implement effective treatments [170,171,172,173,174]. Over the last decade, new psychoactive substances (NPS) have become significantly more prevalent, which can include cannabinoid receptor agonists (SCRAs) [159]. Their action is mainly due to their effect on CB1. However, it should be noted that not all cannabinoids are classified as NPS, e.g., phytocannabinoids found in plants other than cannabis [171,172].

Cannabidiol (CBD) is a non-psychoactive compound with potential antipsychotic properties. Like THC, it is a component of *Cannabis sativa*. Importantly, cannabis consumption is associated with a risk of strong side effects, such as psychiatric disorders, addiction and anxiety. THC contained in cannabis is mainly responsible for these effects. THC exposure can lead to positive symptoms in patients with schizophrenia. This effect may be due to increased dopaminergic transmission in the prefrontal cortex and caudate nucleus [175,176]. It has been shown that cannabis users show marked changes in the limbic system and prefrontal cortex, which are rich in CB1 receptors. Even a single exposure to cannabinoids can cause multiple changes in the CNS, leading to increased release of neurotransmitters, such as dopamine and endogenous opioids [175,177]. A study by McGuire et al. [178] showed that after administration of CBD to patients with schizophrenia, a reduction in the occurrence of positive symptoms of schizophrenia was observed compared to a control sample. However, no improvement in cognitive function in schizophrenia was observed [178]. Also, a study by De Filippis et al. [179] showed that cannabidiol has antipsychotic activity. This effect is associated with the inhibition of the formation of an inflammatory response. Exogenous cannabinoids reduce the secretion of inflammatory mediators, such as pro-inflammatory cytokines and inhibit the activation of microglia cells. Studies comparing the effects of THC and CBD have also been conducted. Interestingly, CBD has been shown to have no psychogenic effects compared to THC and may attenuate THC-induced anxiety [180]. There is evidence to suggest an important role for TRPV1 receptors in the effects of CBD in schizophrenia [181]. Currently, the exact mechanism of cannabidiol’s antipsychotic action is still unclear. It is thought that it may be due to CBD’s ability to inhibit the fatty acid amide hydrolase (FAAH) enzyme which is responsible for deactivating anandamide. This is supported by the results of studies showing increased levels of anandamide in CBD-treated patients [182]. Anandamide levels in the CSF are thought to be inversely related to the severity of psychotic symptoms in schizophrenia. Furthermore, the effects of anandamide may attenuate the transition of mild psychotic symptoms to exacerbated ones [182]. Results from other research teams are not as conclusive. Therefore, CBD is currently only used in very rare cases of schizophrenia. High-quality randomized trials are needed to confirm its efficacy and safety [159,178,181].

## 9. Correlation—Schizophrenia and Depression

According to many scientific reports, depression is a common symptom due to every phase of schizophrenia [183,184]. That negative symptom has become one of the reasons for suicide among people suffering from schizophrenia [184,185,186]. Some researchers have estimated that depressive symptoms are one of the most important risk factors for suicide in the early phase of schizophrenia [187]. Depression can also lead to polypharmacy [188], impaired social functioning [189] and even reduced quality of life. Generally, comorbid depression in schizophrenia often hasn’t been properly diagnosed which doesn’t allow the implementation of its treatment among patients [1,11]. Elucidating the correlation between schizophrenia and depression so as to be able to manage with it is indispensible [190,191]. Among many studies, we could see a discrepancy between the prevalence of comorbid depression in schizophrenia. In 2018 Dai et al. [192], using the 17-item Hamilton Depression Rating Scale (HAMD-17), proved that depression occurred in about 54,6% of patients with schizophrenia. During another study [191] amid outpatients with diagnosed schizophrenia, only about 31% suffered from depression. These discrepancies may have been probably the result of different study settings or assessment instruments [193]. It has been estimated that depression concerns 7% to 75% of patients with schizophrenia [193]. The meta-analysis in 2020 conducted by Li et al. [190] clearly confirmed that comorbidity with depression and schizophrenia is a very common occurrence which may probably depend on some factors like gender, age, duration of schizophrenia, involvement in social life, or even the quality of the study. In 2018, Krynicki et al. [194] created a systematic review about the relationship between negative symptoms and depression in patients suffering from schizophrenia. It appeared that the distinction between negative symptoms and depression in schizophrenia is not so clear-cut, as the two show a close correlation [195]. The symptoms like pessimism, low mood or thoughts about suicide are characteristic more to depression whereas alogia or blunted affect to negative symptoms. While, in both mentioned domains anhedonia, anergia or avolition could be observed. Undoubtedly, awareness of suffering from schizophrenia leads to depressive symptoms, which was in detail analyzed by Ampalam et al. in 2012 [195]. The correlation of depression in the schizophrenic population in the study was presented with Pearson correlation and it was 0.758. These results confirmed earlier reports in medical publications, suggesting that the better insight, the lower mood among schizophrenic patients [196,197]. Moreover, the longer duration of schizophrenia, as well as the age of patients, have favored the rarer occurrence of the symptoms of depression [195]. The quality of life in patients with schizophrenia has also had a significant impact on the occurrence/nonoccurrence of depression. According to Shargh et al. [191], there is a negative correlation between highly advanced depression and low quality of life among these patients. Depression in patients with schizophrenia becomes the cause of their dissatisfaction with life [189]. Furthermore, it has been shown that a reduction in depressive symptoms actually leads to improved life satisfaction (such as social relationships or psychological well-being) [189,198]. Depressive symptoms may also lead to a higher relapse rate, gradual social withdrawal, poorer response to treatment, a tendency to addiction, or reduced cognitive function [189].

Mumtaz et al. [188] analyzed reviews from both clinical and preclinical trials so as to define the effect of social isolation on neuropsychological disorders like depression or schizophrenia [199,200,201].

It has been proven that social isolation causes both behavioral and neurochemical changes. Moreover, this leads to dysregulations in endocannabinoid and dopaminergic systems which are observed in schizophrenia and depression [202]. Cortisol determines the major role in the HPA axis functioning. Cherian et al. [203] investigated the relationship between the level of cortisol and cognition with types of neuronal disorders. Definitely, the higher evening level of the mentioned hormone is observed in patients with major depression than schizophrenia which suggests the dysregulation of the HPA axis in patients with depression. Research suggests that cortisol secretion in patients with schizophrenia increases before an acute episode, which may be an appropriate marker of vulnerability to the disease [195,203,204,205,206].

## 10. Activation of the HPA Axis Due to Environmental/Oxidative Stress as a Risk Factor for Depression and Schizophrenia

Environmental stress is the response of the organism to an environmental stimulus that leads to emotional, cognitive, or behavioral disorders. Environmental stress could be an effect of traumatic life experiences, previous illness, social relations, or perinatal injuries [207,208]. Moreover, the interaction between environmental stressors and vulnerability factors can be a reason for hyperactivity in the nervous system which finally results in the overloading of the area in the brain responsible for processing information and reducing the process of response to the stimuli [207]. Described vicious circle mechanisms thereby cause intensity and manifest symptoms of schizophrenia [207,208]. Even though the connection between environmental stress and neuronal disorders is obvious, the cause itself, despite much previous research, hasn’t been still understood [194]. The impact of stress on the development of many mental disorders is based on common mechanisms connected with higher cortisol levels and the HPA axis [209]. Therefore, it leads to irreversible intracellular changes of plasticity in the synapses by changing the level of BDNF [210]. All types of mechanisms that dysregulated the HPA axis are linked with a higher risk of the development of neuronal disorders [211].

Do et al. [211] demonstrated that oxidative stress contributes to NMDA receptor failure and myelination disorders during nervous system development. Undoubtedly, the effects of many environmental factors (including environmental stress) on the body are associated with transient or prolonged oxidative stress. Schizophrenia is one of the neurodevelopmental disorders that is associated with redox imbalance [212]. The overproduction of reactive oxygen species (ROS), as well as the lack of antioxidants in cells, cause a deficiency of the protective antioxidant glutathione (GSH). These disorders may ultimately lead to the development of schizophrenia [212,213]. In a study by Brown et al. [213], it has been suggested that prevention from some environmental factors can be one of the strategies to cope with schizophrenia. Many schizophrenia’s risk factors affect the development of the fetal brain during pregnancy [214]. As a result of the activation of the maternal immune system, oxidative stress leads to peroxisomal disorders in the fetus [214,215]. Flatow et al. [216] performed a meta-analysis of the associations between oxidative stress and schizophrenia. According to many reviews and clinical trials, several antioxidants have been identified, the levels of which in cells may be a significant biomarker in schizophrenia. The researchers suggested that investigation of the correlation between oxidative stress and clinical features may provide information about the potential mechanisms responsible for the onset of disease. In 2012 Martinez-Cengotitabengoa and colleagues [217] were the first scientists who investigated and described an association between the levels of oxidative stress and neurocognition in patients with first-episode psychosis. The clinical trial confirmed that levels of GSH and executive functions are significantly correlated. Moreover, the perception of oxidative stress as a biomarker and therapeutic target may lead to the discovery of a new treatment of schizophrenia focused on antioxidants [216,218,219]. However, others also argued that oxidative parameters should not be used as one and only schizophrenia biomarkers. In 2014, Gonzalez-Liencres et al. [220] conducted a case-control study with patients suffering from schizophrenia. They discovered that the harmfulness of oxidative stress in schizophrenia is a more complex problem. It occurs that the activity and function of some enzymes involved in antioxidant processes—superoxide dismutase, glutathione peroxidase, or catalase, have not been still clearly understood [221,222]. Gardiner et al. [223] have shown that some neurotrophic factors as neurotrophin 4/5 (NT4/5) plausibly lead to the up-regulation of antioxidants in neurons. It had been suggested that a higher level of NT4/5 is correlated with oxidative stress in neuronal cells, mainly in patients suffering from bipolar disorder [224]. Whereas, both reduced levels of NT4/5 and activity of antioxidant processes have been noted in patients with Alzheimer’s disease [225]. Nevertheless, the association between NT4/5 and schizophrenia remains still unexplained. Probably, the oxidative damage is due to both less cellular protection and magnified oxidative insults. Due to these reports, the oxidative parameters are not specific to cognitive disorders (like schizophrenia) despite their certain potential as markers to determine cognitive faculties [220].

The pathogenesis of schizophrenia and also depression may depend on exposure to oxidative and/or environmental stress. There is ample evidence to suggest that it is environmental stress, mainly related to childhood trauma, which plays a large role in the pathogenesis of depression. Some review papers provide evidence that environmental factors can lead to epigenetic changes in humans [226,227]. This means that a certain stressors can alter gene expression, which can be potentially heritable to the next generation [228]. Park et al. [229] reviewed associations between stress, epigenetics and depression in humans. They suggested that a multidirectional understanding of the correlation between these three compounds may provide the information about pathology, neurobiology and potential new methods for the treatment of depression. According to other findings [230,231], there are two significant markers of oxidative stress where levels are definitely higher in depression—F2-isoprostanes and 8-OHdG. Exposure to stress also increases the production of pro-inflammatory cytokines and other inflammatory mediators (IL-1, IL-6, IFNγ, TNFα, IL-1β) [232]. Interestingly, a study by Kiecolt-Glaser et al. [233] found that children’s exposure to chronic psychosocial stress can significantly reduce their life expectancy (by 7 to 15 years). Another study by Miller et al. [234] proved the increased expression of C-reactive protein (CRP) and IL-6 in women with enhanced predisposition to depression. Long-term exposure to environmental stress (e.g., physical or psychological abuse in childhood) can lead to a sustained increase in the HPA axis activity and the increased CRH release. As a result of increased cortisol release, amygdala neurons become overactive and hippocampal activity is impaired. These permanent changes in the brain increase the body’s vulnerability to stress. Exposure to environmental stressors leads to activation of the sympathetic nervous system and the release of catecholamines [235,236,237,238,239]. The effect of catecholamines on the body is the release of pro-inflammatory cytokines and the decrease in BDNF levels. Pro-inflammatory cytokines are also involved in activation of the HPA axis and they reduce serotonin release [235,236,237,238,239].

**Figure 4:**
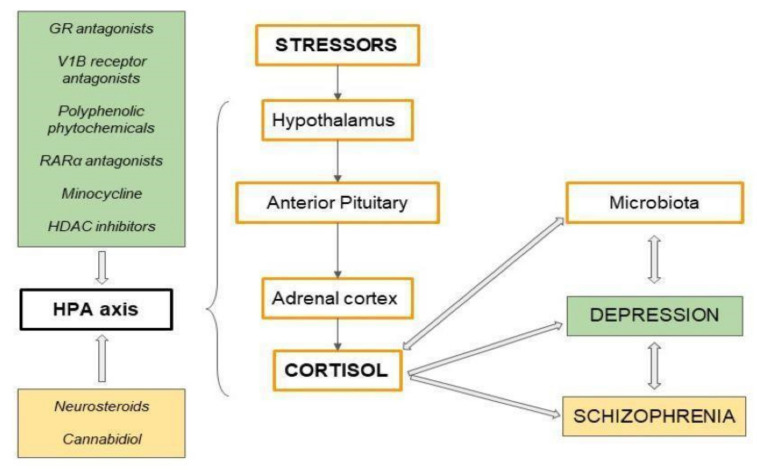
HPA axis in pathomechanisms of depression and schizophrenia: new therapeutic targets affecting the HPA axis.

Neuroinflammation is undoubtedly associated with depression (See Figure 4). This is supported by the fact that the administration of interferon (IFN)-alpha, which is a proinflammatory cytokine, induces depressive symptoms [240,241]. Moreover, proinflammatory cytokines may contribute to the phenomenon of excitotoxicity, associated with excess glutamate in the brain and activation of the kynurenine pathway [242]. These processes result in the formation of active metabolites that interfere with neurogenesis [240,242]. Inflammation may also play a role in the psychosocial changes that often accompany depression [243]. To confirm this, a study was conducted in which participants were exposed to bacterial endotoxin, an inducer of inflammation. Interestingly, not only depressive behaviors but also feelings of social isolation were observed in the participants [243,244]. Inflammation has been shown to affect interpersonal relationships, sensitizing patients to negative and positive social experiences [244]. Therefore, people who suffer from inflammation-related diseases may be at greater risk of loneliness and depression [243,244]. Understanding the role of neuroinflammation in the pathogenesis of depression has led to the development of many new potential treatments for this condition, including anti-inflammatory cytokines whose efficacy has been confirmed in preclinical studies [235,236,237,238,239,245]. Therefore, the discovery of a complex correlation between depression and oxidative stress may allow for a better understanding of its etiology.

## 11. Conclusions

The HPA axis—through its effect on cortisol release—plays a significant role in the pathogenesis of depression. Cortisol levels are significantly elevated in patients with MDD compared to healthy volunteers. High cortisol levels have proved to impair verbal and working memory, thereby reducing cognitive function in depression. The hippocampus, whose volume is significantly reduced in MDD, has been shown to be responsible for HPA axis regulation and neurogenesis [146]. Data from a review of meta-analyses and randomized clinical trials indicate complex links between HPA axis function and serotonergic transmission [23,24]. A review of newer work has also demonstrated HPA axis hyperreactivity in schizophrenia [117,143]. However, it should be noted that for schizophrenia the findings are less conclusive than for depression, and high-quality randomized clinical trials clarifying the exact role of the HPA axis in the pathogenesis of schizophrenia are needed.

This review also highlights the relationship between the gut microbiota and the HPA axis. Stressful events, especially during childhood, can lead to dysbiosis, which may represent an indirect step in the impact of stress on HPA axis function. The link between the gut microbiota and depression is confirmed by the fact that it is involved in the synthesis of serotonin, BDNF and tryptophan metabolism.

A better understanding of the function of the HPA axis in schizophrenia and depression is key to the development of new pharmacological therapies. Cortisol exerts its pharmacological effects by influencing GR and MR. The use of mifepristone, a GR antagonist in depression, has shown the most promising results and the best safety profile. It has also significant benefits in schizophrenic patients, particularly in improving cognitive function. The use of DHEA in schizophrenia has yielded inconclusive results and further research is needed. Studies on the effect of cannabidiol on the development of positive symptoms in schizophrenia seem to be encouraging. More and more data are emerging on the effects of the vasopressin VB1 receptor on the development of depression. These are relatively new findings, so studies are still ongoing to confirm the efficacy of VB1 receptor antagonists in depression but appear to be a promising point of focus.

The mechanism of action of the HPA axis is complex and it is often difficult to determine whether the overactivity of the HPA axis is a cause or a consequence of a disease. Therefore, interfering factors, such as disease duration and severity should be taken into account in further studies. The authors hope that the collected findings will lead to a better understanding of the action of the HPA axis in depression and schizophrenia and will contribute to the development of new pharmacological therapies.

## Data Availability

Not applicable.
